# Seltene Ursache einer bakteriellen Peritonitis

**DOI:** 10.1007/s00117-025-01551-w

**Published:** 2026-01-08

**Authors:** Christopher Kloth, Thomas Breining, Annika Beck, Axel John, Meinrad Beer, Daniel Vogele

**Affiliations:** 1 Klinik für Diagnostische und Interventionelle Radiologie, Universitätsklinikum Ulm, Albert-Einstein-Allee 23, 89081 Ulm, Deutschland; 2 Praxis für Radiologie und Strahlentherapie, Lindau, Deutschland; 3 Institut für Pathologie, Universitätsklinikum Ulm, Ulm, Deutschland; 4 Klinik für Urologie und Kinderurologie, Universitätsklinikum Ulm, Ulm, Deutschland

## Anamnese

Wir berichten über eine 34-jährige Patientin, die sich in der zentralen Notaufnahme unseres Klinikums mit Bauchschmerzen vorstellte. Aus der Vorgeschichte waren eine Spina bifida mit Hemiparese sowie eine ventrikuloperitoneale Shuntanlage vorbekannt. Zudem erfolgte bei der Patientin vor 15 Jahren bei neurogener Dysfunktion der Harnblase ein Blasenhalsverschluss und Anlage eines katheterisierbaren Mitrofanoff-Stomas. Das Mitrofanoff-Stoma ist ein sog. kontinentes Stoma. Dabei wird die Appendix vermiformis als Kanal zur Blasenentleerung zwischen Bauchwand und Blase eingenäht. Für die Urinausscheidung wird mit einem Katheter der Urin mehrmals täglich über das Stoma aus der Blase abgelassen. Im vorliegenden Fall erfolgte die Ausleitung im rechten Unterbauch, worüber die Patientin ihre Blase mittels Einmal-Katheterisierung entleeren konnte. Dies erfolgte in den Tagen vor der Vorstellung laut Patientin subjektiv problemlos.

Laborchemisch zeigte sich eine Leukozytose mit Linksverschiebung sowie eine Kreatinämie. Im U‑Status zeigte sich eine Leukozyturie mit positivem Nitrit. In der initialen Urin- und Blutkultur konnten im Verlauf *Enterococcus faecalis* in signifikanter Keimzahl nachgewiesen werden. Urologisch bestanden anamnestisch vorbekannt eine ausgeprägte Hypertrophie der Harnblase mit einem kranialen Divertikel sowie multiple Konkremente intravesikal.

## Radiologische Diagnostik

Zur weiteren Diagnostik wurde eine Bildgebung mittels kontrastangehobener Computertomographie (CT) des Abdomens in venöser und urographischer Phase zur Eruierung eines Infektgeschehens veranlasst.

**Abb. 1:**
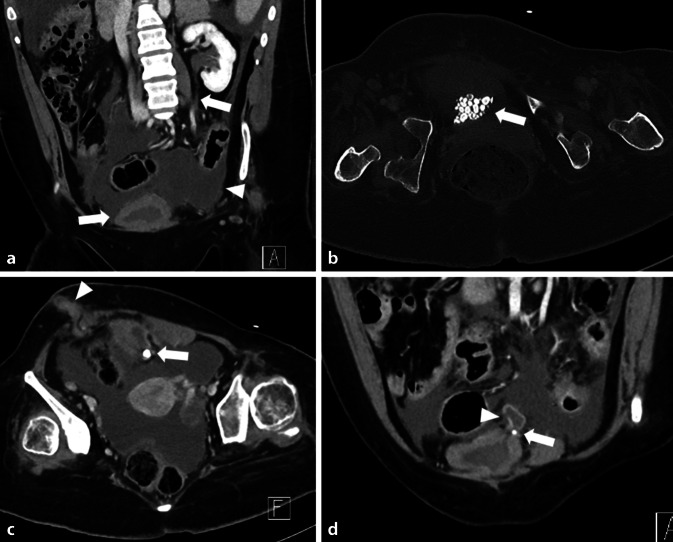
Computertomographie (CT) des Abdomens mit Kontrastmittel in portalvenöser Phase in koronarer (**a**, **d**) und axialer (**b**, **c**) Rekonstruktion (**a**, **c**, **d** Weichteilfenster, **b** Knochenfenster). **a** Es zeigt sich im Unterbauch die zirkulär wandverdickte Harnblase und der kontrastmittelaufnehmende Ureter links (*Pfeile*). Zudem ist reichlich Flüssigkeit im Unterbauch und ein verdicktes, kontrastmittelaufnehmendes Peritoneum zu erkennen (*Pfeilspitze*). **b** Innerhalb der Blase finden sich multiple röntgendichte Konkremente (*Pfeil*). **c** Die Blase zeigt ein nach links kranial gerichtetes Divertikel mit einem darin befindlichen Konkrement (*Pfeil*). Die Ausleitung des Mitrofanoff-Stoma im rechten Unterbauch ist verdickt und nimmt Kontrastmittel auf (*Pfeilspitze*). **d** Abgebildet ist erneut die wandverdickte Blase mit dem nach links kranial gerichteten Divertikel und dem teilweise erfassten Konkrement (*Pfeil*). Die Wand des Divertikels zeigt eine Konturunterbrechung (*Pfeilspitze*)

**Abb. 2:**
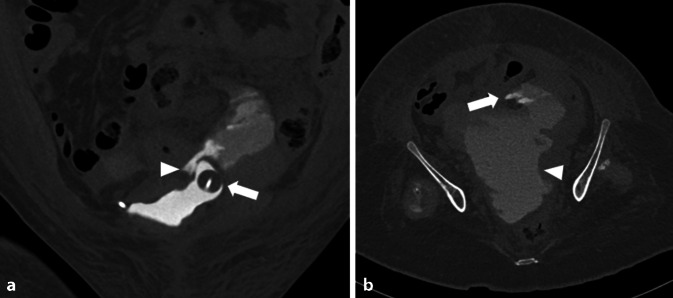
Computertomographie (CT) des Abdomens in urographischer Phase in **a** koronarer und **b** axialer Rekonstruktion (Knochenfenster). **a** Zwischenzeitlich wurde ein Harnblasenkatheter über die Ausleitung im rechten Unterbauch eingebracht. Der geblockte Ballon liegt im Divertikel (*Pfeil*). Zudem Nachweis von austretendem Kontrastmittel/Urin (*Pfeilspitze*). **b** Abzugrenzen ist das austretende Kontrastmittel/Urin mit Ausbildung eines großen Urinoms im Unterbauch (*Pfeilspitze*)

In der durchgeführten CT (Abb. 1) zeigte sich bei ausgeprägtem Aszites eine deutliche Kontrastmittelaufnahme des peritonealen Blattes als Ausdruck einer Peritonitis. Ferner ließen sich multiple Konkremente innerhalb der wandverdickten Harnblase im Sinne einer chronischen Inflammation bzw. bei bekannter neurogener Dysfunktion feststellen. Des Weiteren zeigte sich eine gesteigerte Kontrastmittelaufnahme des Ureters und des Nierenbeckenkelchsystems (NBKS) im Sinne einer diffusen Entzündung. Links kranial war am Dach der Blase eine divertikelartige Aussackung mit Konkrementeinschluss abzugrenzen. Die Wand des Divertikels war zart, kurzstreckig war die Kontinuität der Wand nicht abgrenzbar. Im Verlauf der Ausleitung im rechten Unterbauch zeigte sich ebenfalls eine deutliche Entzündungsreaktion. Zur Darstellung der Abflussverhältnisse wurde eine urographische Phase angefertigt (Abb. 2).

## Wie lautet Ihre Diagnose?

## Definition

Blasenrupturen können in intra- und extraperitoneal sowie spontane Rupturen unterschieden werden. Die Ursachen für eine Blasenruptur mit anschließendem Urinom können hierbei vielfältig sein, wobei Traumata und eine Obstruktion im Verlauf des Harntraktes mit konsekutivem Rückstau als die häufigsten Ursachen vorkommen. Extraperitoneale Rupturen sind in der Regel traumatisch bedingt und stellen insgesamt die häufigste Form dar. Intraperitoneale spontane Blasenrupturen werden in der Literatur vor allem bei Patienten mit vorbestehender struktureller Schädigung der Harnblase beschrieben. Zu diesen zählen Tumoren, Divertikel, chronische Entzündungen oder postradiogene Fibrosen, welche die Harnblase weniger widerstandsfähig gegenüber einer Überdehnung machen und so eine spontane Perforation begünstigen können [1]. Die Blasenkuppel/-dom gilt dabei als Prädilektionsstelle, da die Blasenwand hier am wenigsten widerstandsfähig ist [1]. Intravesikale Konkremente können infolge konsekutiver chronischer Inflammation eine Perforation begünstigen und sind daher als Risikofaktor zu betrachten [2]. Die spontane Ruptur von Harnblasendivertikeln infolge von Konkrementen ist in der Literatur dabei jedoch nur in Form von Einzelfallberichten beschrieben [3, 4]. Im vorliegenden Fall muss daher auch eine akzidentelle traumatische Ruptur infolge der Eigenkatheterisierung diskutiert werden.

Die Resorption von Urin durch das Peritoneum führt zu erhöhten Serumspiegeln des Kreatinins und kann so eine akute Nierenschädigung (AKI) vortäuschen, wie auch im von uns vorgelegten Fall. Die initiale Kreatininämie war am ehesten auf eine peritoneale Resorption des extravesikalen Urins zurückzuführen. Eine akute Nierenschädigung erschien aufgrund der unauffälligen CT-Bildgebung ohne Hinweis auf eine Harnabflussstörung, der raschen Normalisierung der Nierenparameter nach dauerhafter Katheterableitung sowie fehlender klinischer und laborchemischer Zeichen einer prä- oder intrarenalen AKI unwahrscheinlich.

Insbesondere in der direkten postoperativen Situation nach Operationen im Becken, z. B. nach gynäkologischen, visceralchirurgischen oder urologischen Eingriffen, ist ebenfalls an eine mögliche Verletzung der Blase zu denken [5]. Sofern mittels CT in venöser und urographischer Phase die Intaktheit der Harnblase nicht sicher nachgewiesen werden kann, kann zusätzlich über einen transurethralen Katheter eine retrograde Füllung mit verdünntem Kontrastmittel erfolgen [5]. Da es sich bei der spontanen Harnblasenruptur um eine seltene Differenzialdiagnose mit unspezifischer Symptomatik handelt, erfolgt die Diagnosestellung häufig verzögert [6]. Auch im von uns vorgestellten Fall konnte erst durch die mehrphasige CT die Diagnose gesichert werden.

Aufgrund der individuellen Patientenvorgeschichte im oben beschriebenen Fall, mit zahlreichen Katheterisierungen der Blase im Vorfeld, ist dies als weiterer prädisponierender Faktor zu berücksichtigen.

**Diagnose: **Bakterielle Peritonitis/superinfiziertes Urinom bei Ruptur eines Blasendivertikels

Die intraperitoneale Blasenruptur rechtfertigt per se eine zeitnahe chirurgische Sanierung, da sie mit dem Risiko einer Sepsis und Peritonitis einhergeht [7]. Assoziierte kleinere und spontane, nichttraumatische Urinome werden in der Regel konservativ behandelt und resorbieren sich häufig spontan [1]. Ausgedehnte oder superinfizierte Urinome können mittels perkutaner Drainage entlastet werden.

## Therapie und Verlauf

**Abb. 3:**
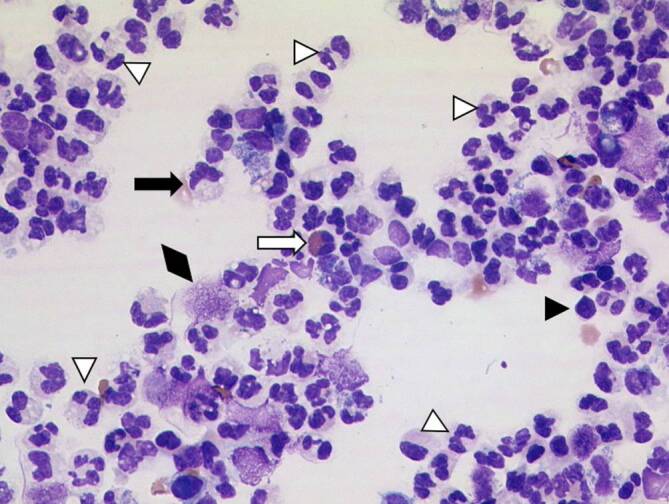
Aszitespunktat mit einem hochfloriden entzündlichen Zellbild mit zahlreichen neutrophilen Granulozyten (*weiße Pfeilspitzen*), einzelnen eosinophilen Granulozyten (*weißer Pfeil*), Lymphozyten (*schwarze Pfeilspitze*), Makrophagen (*schwarzer Pfeil*) und zerfallenden Leukozyten (*schwarze Raute*). Zytospinpräparat, MGG-Färbung, Originalvergrößerung 400:1

Als Therapie erfolgte eine parenterale Antibiose mit Meropenem im stationären Setting. Es erfolgte eine Katheterdauerableitung über das Mitrofanoff-Stoma im rechten Unterbauch, worunter sich der klinische Zustand und die laborchemischen Entzündungsparameter deutlich besserten. Es erfolgte zudem ein Zystogramm, in dem sich die Leckage am Harnblasendivertikel bestätigte. Das abdominelle Urinom wurde mittels Drainage abgeleitet und die gewonnene Flüssigkeit histopathologisch aufgearbeitet. Hierbei zeigte sich ein entzündliches, zellreiches Probenmaterial ohne Anhalt für Malignität (Abb. 3). Zudem konnte auch im Aszites *Enterococcus faecalis* nachgewiesen werden, passend zur Urin- und Blutkultur. *Enterococcus faecalis* ist zwar typischer Bestandteil der Darmflora, wird jedoch bei Patient:innen mit komplexer urologischer Vorgeschichte, insbesondere auch nach Harnblasenaugmentation, häufiger als Erreger von Harnwegsinfektionen nachgewiesen. Die veränderte Anatomie, die Zystolithiasis, die Schleimproduktion des augmentierten Darmsegments sowie intermittierend erhöhte Restharnvolumina begünstigen eine Kolonisation mit *darmtypischen* Bakterien. In unserem Fall ergaben sich keine Hinweise auf eine enterovesikale Fistel: Die CT-Diagnostik zeigte eine freie Perforation ohne Hinweis auf eine Fistel. Zudem bestanden keine gastrointestinalen Symptome sowie keine Fäkalurie.

Da das distale Ende des VP-Shunts innerhalb des Urinoms lag, wurde dieses entfernt und der Shunt temporär infraklavikulär nach extern ausgeleitet. Von einer operativen Sanierung der Blasenruptur wurde trotz bestehender Infektsituation aufgrund der Begleiterkrankungen und dem daraus resultierenden erhöhten operativen sowie Narkoserisiko zunächst abgesehen. Bei im Verlauf fallenden Entzündungsparametern und sistierender Fördermenge über die Drainage konnte diese nach 21 Tagen entfernt und die Patientin nach Hause entlassen werden. Im Verlauf konnte nach unauffälligem Zystogramm auch der Dauerkatheter entfernt und die gewohnte Einmalkatheterisierung wieder aufgenommen werden. Zudem erfolgten eine endoskopische Laserlithotripsie und Evakuation der Blasensteine über das Mitrofanoff-Stoma.

## Fazit für die Praxis

- Harnblasendivertikel sollten mit besonderem Augenmerk hinsichtlich etwaiger Komplikationen betrachtet werden.
- Bei großen Mengen Aszites unklarer Ursache ist differenzialdiagnostisch an ein Urinom zu denken.
- Sofern ein Urinom als Differenzialdiagnose im Raum steht, kann eine urographische Phase diagnostisch hilfreich oder beweisend sein.
